# Heterosis of fitness and phenotypic variance in the evolution of a diploid gene regulatory network

**DOI:** 10.1093/pnasnexus/pgac097

**Published:** 2022-06-29

**Authors:** Kenji Okubo, Kunihiko Kaneko

**Affiliations:** Research Center for Integrative Evolutionary Science, the Graduate University for Advanced Studies, SOKENDAI, Hayama, Kanagawa, 240-0193, Japan; Department of Basic Science, Graduate School of Arts and Sciences, The University of Tokyo, Meguro, Tokyo, 153-8902, Japan; Universal Biology Institute, The University of Tokyo, Bunkyo, Tokyo, 113-0033, Japan; The Niels Bohr Institute, University of Copenhagen, Blegdamsvej 17, Copenhagen, 2100-DK, Denmark

**Keywords:** heterosis, evolution, phenotypic variance, gene regulatory network, sexual reproduction

## Abstract

Heterosis describes the phenomenon, whereby a hybrid population has higher fitness than an inbred population, which has previously been explained by either Mendelian dominance or overdominance under the general assumption of a simple genotype–phenotype relationship. However, recent studies have demonstrated that genes interact through a complex gene regulatory network (GRN). Furthermore, phenotypic variance is reportedly lower for heterozygotes, and the origin of such variance-related heterosis remains elusive. Therefore, a theoretical analysis linking heterosis to GRN evolution and stochastic gene expression dynamics is required. Here, we investigated heterosis related to fitness and phenotypic variance in a system with interacting genes by numerically evolving diploid GRNs. According to the results, the heterozygote population exhibited higher fitness than the homozygote population, indicating fitness-related heterosis resulting from evolution. In addition, the heterozygote population exhibited lower noise-related phenotypic variance in expression levels than the homozygous population, implying that the heterozygote population is more robust to noise. Furthermore, the distribution of the ratio of heterozygote phenotypic variance to homozygote phenotypic variance exhibited quantitative similarity with previous experimental results. By applying dominance and differential gene expression rather than only a single gene expression model, we confirmed the correlation between heterosis and differential gene expression. We explain our results by proposing that the convex high-fitness region is evolutionarily shaped in the genetic space to gain noise robustness under genetic mixing through sexual reproduction. These results provide new insights into the effects of GRNs on variance-related heterosis and differential gene expression.

Significance Statement Heterosis, higher fitness in hybrid populations than in inbred populations, is a long-standing mystery in genetics, evolution, and breeding studies. Heterosis does not necessarily result from a trait determined by a single gene, but likely involves stochasticity and interactions among genes. Through numerical evolution of a gene regulatory network, we demonstrate that heterosis is shaped by evolution to achieve robustness against noise with genetic mixing by sexual recombination in gene expression dynamics. In other words, a mixed population is more robust to noise than an inbred population, as previously revealed experimentally by reduced phenotypic variance. This observed link between heterosis and phenotypic robustness, Mendelian dominance, and the convex single-humped fitness landscape represents a novel avenue in evolution and genetics research.

## Introduction

Sexual reproduction generally involves mixing genetic information from multiple parents, which is a strategy that has been adopted for a wide range of species. Many diploid organisms undergo meiosis, which results in the recombination of two parental genomes to produce gametes, and then the gametes combine to produce the genome of the offspring.

One of the most remarkable phenomena in sexual reproduction among populations is heterosis, whereby hybrid populations exhibit higher fitness than inbred populations obtained by repeated sexual reproduction among genetically close individuals. Inbreeding depression is often regarded as two sides of the same phenomenon of heterosis, which indicates lower fitness of the inbred than hybrid population. To date, the origin of both heterosis and inbreeding depression has largely been discussed from a genetics perspective (1–4), converging on a similar argument.

One of the simplest models proposed to explain heterosis and inbreeding depression is the dominance model based on Mendelian dominant inheritance pattern (5). In Mendelian dominance (6), if two diploid parent genomes have the genes *AA* and *aa*, and show the corresponding traits “A” and “a,” then the child has the gene *Aa* and shows trait “A” when trait “A” is dominant and trait “a” is recessive. In the dominance hypothesis, mutations accumulated in homozygotes of an inbred population are considered to be deleterious and recessive. However, Mendelian dominance cancels out this deleterious mutational effect in heterozygotes of a hybrid population, contributing to its higher fitness. This explanation corresponds to complementation among products from alleles at the molecular level. Although the dominance or complementation model can effectively explain several observations of inbreeding depression, it cannot explain some observations related to heterosis and inbreeding depression (7). First, inbreeding with selection could not reduce the effect of heterosis, although the deleterious and recessive mutations would be decreased in the course of inbreeding with selection, which should then reduce heterosis according to the dominance or complementation model (8, 9). Second, in tetraploids, the deleterious and recessive mutational effect should be reduced according to the dominance model; however, tetraploid organisms do not show less heterosis even though tetraploid inbreeding decreases the ratio of homozygotes (*AAAA* or *BBBB*) (10–13).

In contrast, the overdominance hypothesis has been proposed, in which heterozygotes can acquire a novel trait that confers higher fitness relative to that of both parental homozygotes (5). In the (positive) overdominance model, the heterozygote *Aa* has higher fitness than *AA* and *aa*. Although there is sparse direct evidence for the overdominance model, it can explain some results that cannot be explained by the deleterious and recessive mutation models. For example, the abovementioned increase in heterosis in the artificially evolved species (8, 9) or tetraploid (10–13) can be explained by the overdominance model. At the molecular level, the interaction between alleles is assumed to produce such heterozygote advantage (7). By noting that a quantitative trait studied in the context of heterosis is a result of multiple, potentially interacting, loci or genes (14,15), the relevance of an interaction effect as a result of gene regulatory hierarchies has been proposed (16). For example, Bar-Zvi et al. (17) focused on two factors, i.e. enhancement of growth pathways and impairment of growth inhibition, to explain relatively higher hybrid fitness. For the latter factor, they pointed out the existence of an incompatibility gene regulatory network (GRN), which is suppressed in the heterozygote, leading to heterosis (18).

Another concept that has been proposed to explain this phenomenon is differential gene expression, which is related to overdominance, and has attracted substantial research attention in the context of studying inbreeding depression and GRNs (19–21). In the differential gene expression model, heterozygous F1 hybrids exhibit different gene expression patterns from those of their inbred parents (22) (In contrast to the overdominance model, the different expression pattern itself does not necessarily confer higher fitness.). The existence of differential gene expression and heterosis have shown a positive correlation (15, 23); however, the mechanism explaining this apparent connection has not yet been elucidated.

The traditional arguments related to heterosis described above are mainly based on an assumption of a simple genotype–phenotype relationship; that is, the case in which one gene is associated with one phenotype (6, 8,24). However, recent studies demonstrate the relevance of interactions among genes to determine the ultimate phenotype (25–30). Therefore, it is necessary to construct a model in which the phenotype is determined as a result of genetic interactions to unveil the relevance of the genotype–phenotype relationship to heterosis with respect to fitness and phenotypic variance. Toward this end, we focused on the GRN model (31–36). In a GRN, protein expression depends on the mutual activation or suppression of genes, where a phenotype (e.g. expression of a single protein) depends on the expression levels of multiple genes (31). Recent transcriptome analyses have revealed such interactions among genes (37). Furthermore, interactions of gene regulatory regions (i.e. promoters, transcription factor binding sites, and enhancers) change more frequently throughout the evolutionary course compared with changes in protein-coding genes (38).

The GRN model itself assumes neither dominance nor overdominance. Therefore, there is no apparent Mendelian dominance with respect to gene expression levels in a diploid-GRN model, and whether or not such dominance can emerge through evolution is not evident based on the model alone (36). Although some studies have addressed heterosis using GRN models (39,40), the relevance of phenotypic robustness to noise and genomic mixing by sexual recombination to heterosis has not yet been considered in this context.

Hence, studying how phenotypic variance changes between hybrid and inbred populations is also important. Indeed, in contrast to the standard context of heterosis, in which the fitness or a fitness-related trait is compared between inbred and hybrid populations, Phelan and Austad (41) examined data relating to the traits of over 100 species, and demonstrated that the variance of traits also shows heterosis; that is, the variance of traits for hybrid populations is smaller than that of inbred populations. Furthermore, gene expression dynamics generally involve stochastic noise either due to environmental fluctuation or internal stochasticity in organisms, and the response against such noise is determined by the genotype corresponding to the GRN. However, the possible link between noise and heterosis in a GRN has rarely been discussed (42), especially from the perspective of quantitative genetics. Nevertheless, stochasticity in phenotypes (i.e. gene expression levels) and noise robustness have recently received substantial attention in the context of homeostasis, cell state selection, and GRNs (26,43–49). Considering the result of Phelan and Austad (41), it is important to explore the possibility of the heterosis of noise-induced phenotypic variance, because total phenotypic variance contains the non-negligible contribution of noise-induced phenotypic variance (50).

In this study, we analyzed heterosis and variance-related heterosis by simulating the evolution of a diploid-GRN. The organization of this paper is as follows. First, we introduce the diploid-GRN model and its evolutionary simulation. Second, we compare the fitness of homozygote and heterozygote groups generated from evolved populations and the outcome of fitness-related heterosis. Third, we compare the phenotypic variances between homozygote and heterozygote groups, demonstrating that the variance in the latter is markedly reduced. Additionally, we compare the ratio of heterosis related to phenotypic variance obtained in our simulation to previous measurement data (41). We also investigate the correlation between hybrid fitness and the dominance of gene expression patterns or differential gene expression that emerged through evolution. Finally, an intuitive explanation of the results is provided regarding the fitness landscape.

## Description of the model and simulation

### Evolution simulation based on a diploid-GRN model

First, we introduce the theoretical GRN model, which is largely based on the model presented in (36). Each gene *i*( = 1, 2,..., *N*) has an expression level *x_i_*(*t*) at time step *t*. In the model, *x_i_*(*t*) is scaled such that it takes on a value between zero (nonexpression) and one (expression). Each gene interacts with other genes and with itself, with interaction between the *j*th gene and the *i*th gene described by the matrix *J_ij_* (31–35). *J_ij_* can take three values: +1, −1, or 0, which represent the activation, inhibition, and lack of interaction between gene *i* and gene *j*, respectively. We adopted a discrete-time model (31,44, 51,52), in which the expression level *x_i_*(*t* + 1) in the next time step is determined by $\sum _{j =1}^{N}J_{ij}x_j(t)$, which represents the gene regulatory effect. According to the sigmoid function *f*[*x*] = 1/(1 + exp [ − β*x*]) with a large β( = 100), the expression dynamics of the haploid, where the individual has one set of genome, is given by

(1) \begin{equation*} x_i(t+1)=f[\sum _{j=1}^N J_{ij}(x_j(t)-\theta )], \end{equation*}

where *θ* is the threshold for the expression level.

Therefore, we extended this model to the diploid context to investigate heterosis. We supposed that the products from each chromosome are indistinguishable since promoter or enhancer evolution is faster than that of the coding gene regions (38). Therefore, we can redefine *x_i_*(*t*) as an average of two expression levels in two genomes. Since diploid cells have two regulatory matrices, $J^{(1)}_{ij}$ and $J^{(2)}_{ij}$, the averaged expression level *x_i_*(*t*) affects the promoter or enhancer of two genomes, 1 and 2. The gene expression dynamics of a diploid-GRN are then determined by the sum of two indistinguishable products from two chromosomes; thus, the dynamics are given by modifying $f[\sum _{j=1}^N J_{ij}(x_j(t)-\theta )]$ to $\frac{1}{2}(f[\sum _{j=1}^{N} J^{(1)}_{ij}(x_j(t)-\theta )] + f[\sum _{j=1}^{N} J^{(2)}_{ij}(x_j(t)-\theta )])$ as

(2) $$\begin{eqnarray*}
x_i(t+1) =\frac{1}{2}(f[\sum _{j=1}^{N} J^{(1)}_{ij}(x_j(t)-\theta )]
\end{eqnarray*}
$$

(3) $$\begin{eqnarray*}
+ f[\sum _{j=1}^{N} J^{(2)}_{ij}(x_j(t)-\theta )]) + \sqrt{x_i(t)}\eta (0, \sigma ).
\end{eqnarray*}
$$

(See the Materials and Methods for details of derivation.)

In (3), Gaussian noise, $\sqrt{x_i(t)}\eta (0,\sigma )$ of small magnitude, is added to the dynamics to account for the stochasticity in gene expression. The noise term increases $\sqrt{x_i(t)}$, as is often adopted in stochastic gene expression dynamics. (This could avoid *x_i_*(*t*) crossing over to a negative value for a continuous time model; however, for a discrete-time model, such crossing could happen, in which case *x_i_*(*t*) is reset to zero.)

The calculation of the dynamics is based on the following settings. The computation starts from the given fixed initial expression levels {*x_i_*(0)}, where *x_i_*(0) = 0 for most genes and *x_i_*(0) = 1 for several genes. *x_i_*(*t*) reaches a fixed point after a certain time *T* in most cases, where *x_i_*(0) = 0, *x_i_*(0) = 1, or *x_i_*(0) = 0.5 is achieved depending on the structure of the network *J_ij_* in the diploid-GRN model. However, *x_i_*(0) = 0.5 is hardly ever reached for the evolved GRN under the following fitness definition. The noise term $\sqrt{x_i(t)}\eta (0,\sigma )$ kicks the state out of a certain fixed point, although the system is usually attracted to the same fixed point or to some other point.

Evolutionary simulation of the diploid-GRN was carried out according to the following procedure, using a meiosis-like genetic algorithm. First, fitness is determined by the distance of the expression pattern of genes {*x_i_*(*T*)} from a prescribed target pattern $x^\mathrm{target}_i=1$ for *i* = 1, 2,..., *M*( < *N*). In this simulation, *M* = 10 and *N* = 100. Only some genes have this target pattern, so that the expression levels of most genes are not under selection pressure. That is, there is no preference between *x_i_*(0) = 0 or *x_i_*(0) = 1 for most genes. Next, genetic change is introduced into genomes. After the fitness computation, two parents are chosen with a probability exponential to the fitness. A total of two genomes in each parent, $J^{(1)}_{ij}$ and $J^{(2)}_{ij}$, are mixed by recombination to provide a gamete. Next, two gametes from the two parents provide a new $J^{(1)}_{ij}$ and $J^{(2)}_{ij}$ in the next generation. After meiosis, the elements of the new $J^{(1)}_{ij}$ and $J^{(2)}_{ij}$ are modified by a mutation with the rate μ. This mutation is included as a change in the matrix element *J_ij_* to 0 or ±1 other than *J_ij_* itself with equal probability (see Materials and Methods for details on the evolution procedure).

### Defining homozygote and heterozygote groups

The numerical procedure for generating the homozygote and the heterozygote groups was as follows. We prepared the genome population at a particular generation in the evolutionary simulation. First, we extracted 2*K* genomes $J^{\lt k\gt }_{ij}(k=1....2K)$ from *K* diploid individuals in the evolutionary simulation. We then made a complete copy for each 2*K* genome, creating a population of 2*K* complete homozygotes (e.g. $J^{\lt k\gt }_{ij}J^{\lt k\gt }_{ij}$), which is defined as the homozygote group(The dynamics of the complete homozygote are identical to those of the $J^{\lt k\gt }_{ij}$ haploid.). Next, two different genomes, $J^{(1)}_{ij}$ and $J^{(2)}_{ij}(\ne J^{(1)}_{ij})$, were randomly chosen from 2*K* genomes $J^{\lt k\gt }_{ij}(k=1....2K)$ as heterozygotes, and such 2*K* heterozygotes constituted the heterozygote group. Note that this procedure is not entirely equivalent to the *evolution* of the homozygote group or the heterozygote group.

This procedure corresponds to an extreme case of heterosis for long inbreeding generations over many inbreeding populations. One might also expect that such generation of a pure homozygote group would be rare in a natural population with a low mutation rate and would be accompanied by inbreeding depression. Therefore, we also examined the population separation procedure by separating the evolved population into two groups, and conducted the same evolutionary simulation for each group separately to compare our results with the usual experiments of heterosis or inbreeding depression.

We first measured the fitness of the homozygote and heterozygote groups, where the mean values for the groups are given by *W*^homo^ and *W*^hetero^, respectively. Next, phenotypic variance due to noise (*V*_noise_) is defined as the magnitude of the variance of *x_i_*(*T*) for individuals with the same genotype but with different levels of noise added to the gene expression dynamics (see the Material and Methods for the definition). To eliminate the effect of the mean phenotype, the coefficient of variation (*CV*_noise_), which is the standard deviation($\sqrt{V_\mathrm{noise}}$) divided by the mean value ($\bar{x_i}$), was compared. *CV*_noise_ is computed as the average of the CV over genes *i*( = 1...*N*) for the homozygote and heterozygote groups, giving $CV^\mathrm{homo}_\mathrm{noise}$ and $CV^\mathrm{hetero}_\mathrm{noise}$, respectively.

## Results

We conducted the simulations 30 times with different seeds for random numbers for each parameter set. Each of the 30 runs of the simulation is referred to as a “realization” herein.

### Fitness-related heterosis

**Fig. 1.**
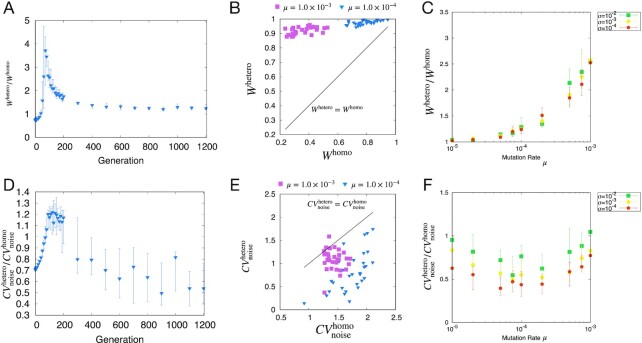
Evolution, correlation, and mutation-rate dependence of heterosis. (A) Change in the degree of fitness-related heterosis *W*^hetero^/*W*^homo^ throughout evolution, calculated for 30 realizations in each generation. Points represent the median, and error bars represent the range between the first and third quantiles. *W*^hetero^/*W*^homo^ increased initially and then decreased. The mutation rate per edge was μ = 1.0 × 10^−4^ and the noise strength was σ = 1.0 × 10^−4^. (B) Correlation between *W*^hetero^ and *W*^homo^ for each realization. The noise was set to σ = 1.0 × 10^−4^. Even under the same conditions, variation occurred in *W*^hetero^ and *W*^homo^. For all realizations in both conditions, the relationship *W*^hetero^ > *W*^homo^ was maintained despite realization-to-realization variation. (C) Increase in *W*^hetero^/*W*^homo^ with mutation rate μ. Points represent the median, and error bars represent the range between the first and third quantile for 30 realizations for different noise magnitudes σ. The ratio *W*^hetero^/*W*^homo^ increased with μ and *W*^hetero^/*W*^homo^ > 1 was maintained. (D) Evolution of the median value of the degree of variance-related heterosis, $CV^\mathrm{hetero}_\mathrm{noise}/CV^\mathrm{homo}_\mathrm{noise}$. The $CV^\mathrm{hetero}_\mathrm{noise}/CV^\mathrm{homo}_\mathrm{noise}$ increased initially and then decreased through evolution, reaching a median value of approximately 0.4 (parameters and conditions were same as in A). (E) $CV^\mathrm{homo}_\mathrm{noise}$ and $CV^\mathrm{hetero}_\mathrm{noise}$ correlation for each realization (parameters and conditions were same as in B). The relationship $CV^\mathrm{homo}_\mathrm{noise}\gt CV^\mathrm{hetero}_\mathrm{noise}$ was maintained despite realization-to-realization variation. (F) Change in $CV^\mathrm{hetero}_\mathrm{noise}/CV^\mathrm{homo}_\mathrm{noise}$ with μ. $CV^\mathrm{hetero}_\mathrm{noise}/CV^\mathrm{homo}_\mathrm{noise}$ increased with μ in this range.(Parameters and conditions were the same as in C).

First, we discuss the case of heterosis related to fitness. The change in the degree of fitness-related heterosis *W*^hetero^/*W*^homo^ during evolution is shown in Fig.1(A). The ratio of *W*^hetero^/*W*^homo^ increased for earlier generations and then decreased. After evolution (at the 10,000th generation), inequality *W*^hetero^/*W*^homo^ > 1 was maintained; therefore, fitness-related heterosis was broadly observed. (See Figure S1 (Supplementary Material) for the evolution of the distributions of *W*^homo^ and *W*^hetero^.)

In Fig.1(B), we plotted *W*^hetero^ versus *W*^homo^ for each realization after evolution. *W*^hetero^ > *W*^homo^ was satisfied, indicating the evolution of heterosis. Then, across each realization, we computed the average ratio of *W*^hetero^/*W*^homo^ and examined its dependence on the mutation rate. As shown in Fig1(C), *W*^hetero^/*W*^homo^ increased with μ, indicating that a greater degree of heterosis evolved at a higher mutation rate. Additionally, at higher μ, the ratio of heterosis *W*^hetero^/*W*^homo^ was larger at a larger σ.

### Variance-related heterosis

The evolution of the mean value of the degree of variance-related heterosis $CV^\mathrm{hetero}_\mathrm{noise}/CV^\mathrm{homo}_\mathrm{noise}$ is shown in Fig. 1(D) (see Figure S2 (Supplementary Material) for the evolution of the distributions of $CV^\mathrm{homo}_\mathrm{noise}$ and $CV^\mathrm{hetero}_\mathrm{noise}$). Note that we regard the lower *CV*_noise_ value as an advantage as it indicates higher robustness to noise; in this case, noise is reduced by the gene expression dynamics to achieve a stable target pattern. Therefore, the lower $CV^\mathrm{hetero}_\mathrm{noise}/CV^\mathrm{homo}_\mathrm{noise}$ value shows larger heterosis. $CV^\mathrm{hetero}_\mathrm{noise}/CV^\mathrm{homo}_\mathrm{noise}$ increased from 0.5 initially and then decreased to approximately 0.4 after evolution (at the 10,000th generation). For a random network at the first generation, the estimated ratio is 0.5 (see Supplementary Material Appendix). Because such a random network would be rare in an evolved population, a ratio less than one indicates nontrivial variance-related heterosis. The variance-related heterosis in the random network is generated by the existence of *x_i_* = 0.5 as a fixed point in the heterozygotes; however, the variance-related heterosis in the evolved population is not derived from that fixed point because heterozygotes achieved the *x_i_* = 0 or *x_i_* = 1 state after evolution and had a more robust expression pattern than that of the homozygotes (see Figure S3, Supplementary Material). We also computed the frequency of the expression levels in the homozygote and heterozygote groups. As shown in Figure S3 (Supplementary Material), the frequency in the heterozygote group is almost zero or one, whereas the frequency of taking on intermediate values is higher in the homozygote group. This difference leads to variance-related heterosis and the higher robustness in the heterozygote group.

As shown in Fig. 1(E), we measured $CV^\mathrm{homo}_\mathrm{noise}$ and $CV^\mathrm{hetero}_\mathrm{noise}$ after evolution for different realizations. In all realizations, $CV^\mathrm{homo}_\mathrm{noise}\gt CV^\mathrm{hetero}_\mathrm{noise}$ was maintained, which indicates that the noise-induced variance of the heterozygotes was smaller than that of the homozygotes. The dependence of $CV^\mathrm{hetero}_\mathrm{noise}/CV^\mathrm{homo}_\mathrm{noise}$ on μ is shown in Fig. 1(F), demonstrating $CV^\mathrm{hetero}_\mathrm{noise}/CV^\mathrm{homo}_\mathrm{noise}\ \lt 1$ for all μ and the ratio increased with μ. For μ > 1.0 × 10^−4^, $CV^\mathrm{hetero}_\mathrm{noise}/CV^\mathrm{homo}_\mathrm{noise}\ \lt 0.5$ was obtained after evolution, showing higher variance-related heterosis than obtained for the case of random networks. At higher μ, the ratio of heterosis $CV^\mathrm{hetero}_\mathrm{noise}/CV^\mathrm{homo}_\mathrm{noise}$ was higher at a larger σ.

### Comparison with previous measurement data

By measuring the variance of traits from data of 172 traits drawn from 15 different species, Phelan and Austad (41) reported that hybrid populations exhibited fewer phenotypic fluctuations than inbred populations. These data were explained by the authors (41) as follows: *“Data covering a broad range of taxa, including invertebrate and vertebrate animals as well as plants, demonstrate that the assumption that genetic variability and environmental variability are independent of each other is unwarranted.”* The values listed are the phenotypic standard deviation of the inbred population and the crossbred population divided by the mean (CV).

**Fig. 2.**
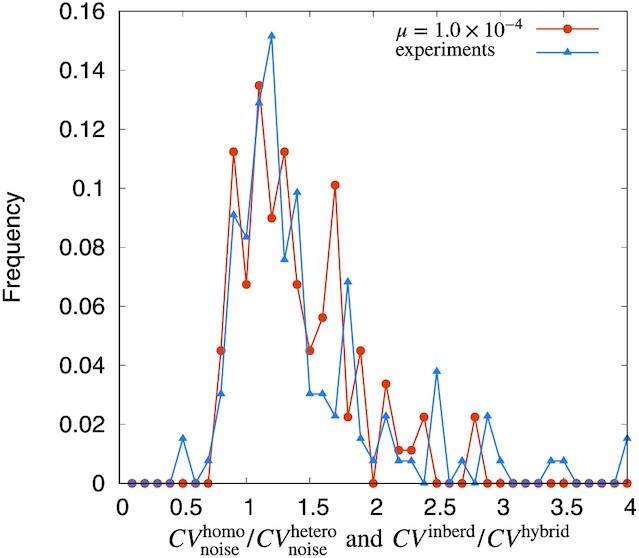
Comparison of the heterosis obtained with the present simulations and previous experimental data (41). Distribution of the ratio of noise-induced phenotypic CV $CV^\mathrm{homo}_\mathrm{noise}/CV^\mathrm{hetero}_\mathrm{noise}$ for our model and previous experimental data (*CV*^inbred^/*CV*^hybrid^). The simulation results (orange dots) are for μ = 1.0 × 10^−4^ and the σ range for [10^−4^, 10^−2^], whereas the measurement data for *CV*^inbred^/*CV*^hybrid^ were obtained for inbred and crossbred populations (blue triangles) from (41). The number of realizations is set to 30 in this figure.

We plotted the distribution of $CV^\mathrm{homo}_\mathrm{noise}/CV^\mathrm{hetero}_\mathrm{noise}$ for a low mutation rate in our simulation (Fig.2). In this case, *CV*_noise_ and the total phenotypic CV were positively correlated, as also shown in previous studies (50,53–56). Note also that the ratio $CV^\mathrm{homo}_\mathrm{noise}/CV^\mathrm{hetero}_\mathrm{noise}$ has been inverted from that used in the previous section to more clearly emphasize the comparison for the region $CV^\mathrm{homo}_\mathrm{noise}\gt CV^\mathrm{hetero}_\mathrm{noise}$.

As shown in Fig. 2, $CV^\mathrm{homo}_\mathrm{noise}/CV^\mathrm{hetero}_\mathrm{noise}$ was similar to the previous experimental data. These results imply that the variance-related heterosis obtained by the diploid-GRN model would be consistent with field data from actual populations.

### Correlation of heterosis with pattern dominance and differential gene expression

In this subsection, we discuss how heterosis is correlated with dominance and differential gene expression, as addressed in (8,22, 24). Here, we need to extend the definition of dominance to study the collective gene expression in the GRN rather than considering the expression of only a single gene. Thus, we defined and computed pattern dominance (36)(The pattern dominance also takes into consideration the expression of nontarget genes, even though it does not directly relate to the fitness. We have discussed the relevance of the expression pattern dominance to fitness in our previous paper (36). The expression pattern dominance is correlated to the robustness against noise, which is also correlated to fitness. Thus, fitness, dominance, and robustness are highly interconnected (36)) and differential gene expression from the gene expression patterns and explored their correlation with heterosis.

To introduce the dominance of gene expression patterns, we defined and computed pattern dominance according to the following procedure. We first computed the gene expression patterns over a group of genes for given homozygotes *AA* and *BB* taken from an evolved population, which is represented by $x^{AA}_i$ and $x^{BB}_i$. Next, we computed the gene expression pattern of heterozygotes *AB*, given by $x^{AB}_i$. For genes satisfying $x^{AA}_i \ne x^{BB}_i$(as a numerical contribution, $|x^{AA}_i-x^{BB}_i|\gt 0.1$ is adopted(In the evolved population, almost all genes have *x_i_* = 0 or *x_i_* = 1 expression for maintaining fitness as a network.)), we examined whether the expression pattern of the heterozygotes is biased toward one of the homozygote expression patterns such as $x^{AB}_i\simeq x^{AA}_i$(as a numerical contribution, $|x^{AB}_i-x^{AA}_i|\lt 0.1$ is adopted) or $x^{AB}_i\simeq x^{BB}_i$(as a numerical contribution, $|x^{AB}_i-x^{BB}_i|\lt 0.1$ is adopted), and computed the ratio of the number of genes, $x^{AB}_i\simeq x^{AA}_i$ or $x^{AB}_i\simeq x^{BB}_i$. Pattern dominance is defined by the larger of the two ratios. If the expression of each gene is independent, such bias is not expected, and the pattern dominance is 0.5. The pattern dominance is 1.0 if $x^{AB}_i\simeq x^{AA}_i$ or $x^{AB}_i\simeq x^{BB}_i$ for all *i* with $x^{AA}_i\ne x^{BB}_i$.

The ratio of differential gene expression was also computed to investigate the correlation between heterosis and differential gene expression, as discussed in earlier studies (19–22). For a set of genes with $x^{AA}_i\simeq x^{BB}_i$, the ratio of genes *i* of heterozygotes, satisfying $x^{AB}_i\ne x^{AA}_i(\simeq x^{BB}_i)$, is computed, which gives the degree of differential gene expression (see the Methods section for a detailed description of pattern dominance and differential gene expression calculations).

Here, we remark on the definition of pattern dominance. (Mendelian) dominance is generally defined regardless of fitness. Trait “A” is dominant when *Aa* shows trait “A” even though its fitness is lower than that of “a.” Following this, the definition of pattern dominance adopted herein is also independent of fitness. The expression of nontarget genes(In the present model, the fitness is not assigned for nontarget genes, and they are (quasi)neutral as for *x_i_*(0) = 0 or *x_i_*(0) = 1. Therefore, whether or not the gene is expressed directly concerns the fitness) in our model, the major aspect of the pattern dominance and differential gene expression pattern is not directly related to fitness. This is in contrast to the “dominance model” for heterosis, which assumes deleterious and recessive mutation. Therefore, our model’s higher pattern dominance value does not directly support the dominance model. The definition of overdominance as well as underdominance is also based on fitness. Hence, the differential gene expression in our model does not necessarily support the overdominance model either.

**Fig. 3.**
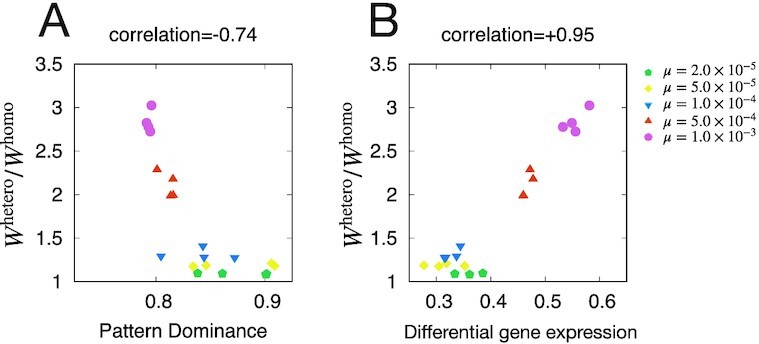
Correlation between heterosis and dominance (differential gene expression). (A) Correlation between pattern dominance and *W*^hetero^/*W*^homo^. (B) Correlation between differential gene expression and *W*^hetero^/*W*^homo^. Each point corresponds to different mutation rates denoted by different symbols, whereas different points correspond to different noise magnitudes σ of [1 × 10^−4^, 1 × 10^−2^]. Points represent the average for the 10,000th generation out of 30 realizations.

The correlation between pattern dominance, differential gene expression, and fitness-related heterosis is shown in Fig.3 (see Figure S4 (Supplementary Material) for the correlation with variance-related heterosis). Heterosis *W*^hetero^/*W*^homo^ exhibited a negative correlation with pattern dominance but a positive correlation with differential gene expression. From these results, we could explain the correlation between differential gene expression and heterosis, as is also discussed in (19–22). In contrast, the dominance of expression pattern was not supported (note that since we did not define deleterious mutation, this does not entirely exclude the possibility of the dominance model). Contrastingly, variance-related heterosis was positively correlated with the pattern dominance but negatively correlated with differential gene expression; however, the absolute values of correlation coefficients were smaller than that for fitness-related heterosis (see Figure S4, Supplementary Material).

The frequencies of the expression levels in the homozygote and heterozygote groups are shown in Figure S3 (Supplementary Material). In the homozygote group, the opposite expression sometimes appears. Therefore, this simulation is reasonable for exploring pattern dominance or differential gene expression.

### Explanation of the results according to the convex fitness region and single-humped fitness function

This section proposes an intuitive explanation of our results with visualization of the fitness landscape. At present, this remains a hand-waiving argument, and further mathematical elucidation and experimental verification are required in the future for validation.

**Fig. 4.**
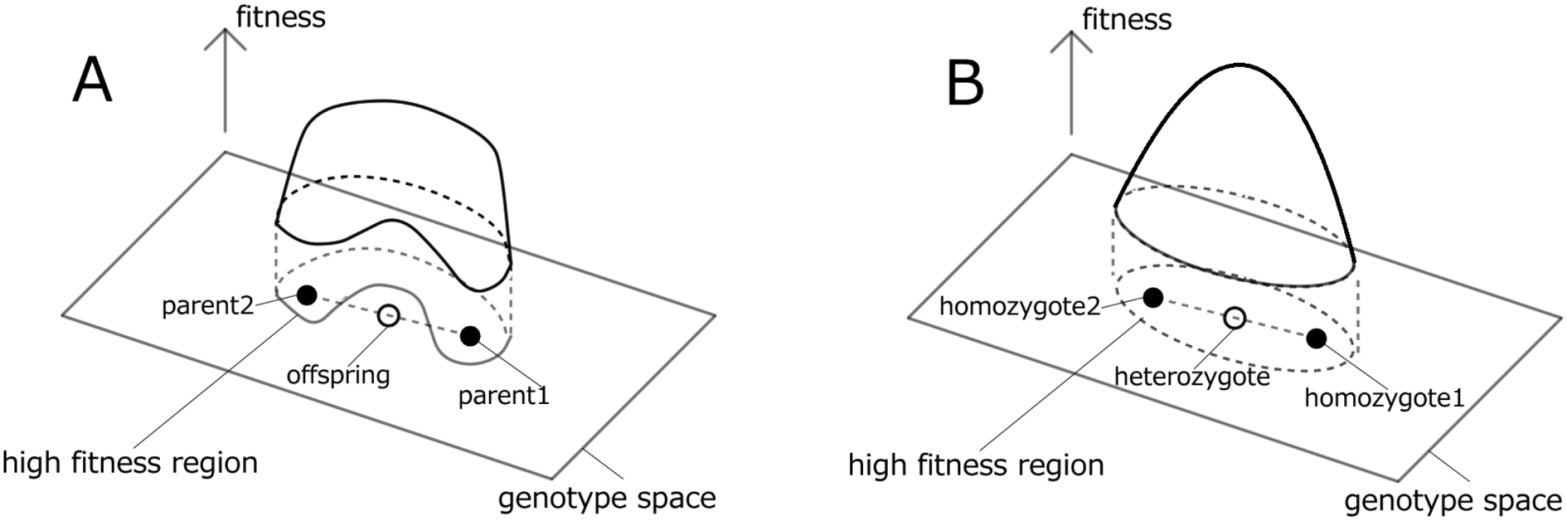
Schematic of the fitness landscape and high-fitness region. (A) When the high-fitness region around the genome population is not convex, the offspring may have lower fitness than the parents; therefore, the lineage will not be selected. (B) When the high-fitness region around the genome population is convex over the genotype space, the offspring’s fitness is higher than or equal to that of the parents taken at the periphery of the convex fitness landscape or those generated from them by mutation only. Here, even if the average fitness remains constant, there are still variations in genomes, and some heterozygotes with lower fitness may exist, which are placed in the periphery of the convex region. If we consider them homozygotes, the fitness will be lower than that of heterozygotes. Here, when we chose a pair from the opposite side of the periphery to demonstrate the relevance of the convexity to robustness, the fitness of the heterozygote from them is higher than that of the homozygote, which provides one reason for heterosis.

We consider a fitness landscape over a genotype space (57). In particular, we focus on the high-fitness region. After evolution, offspring are produced from the two parents by meiosis within this high-fitness region. The genotypes of the diploid genome $(J^{(1)}_{ij}+J^{(2)}_{ij})/2$ will be located approximately at the internal division of the genotype of the parents(The heterozygote genome is not precisely represented by $(J^{(1)}_{ij}+J^{(2)}_{ij})/2$; however, we use this approximate description for simplicity). The offspring must have high fitness; otherwise, the offspring will not be selected. For example, when the higher-fitness region around the genome population is not convex (Fig.4A), the offspring may not be selected because of their lower fitness compared with that of the parents. Therefore, the high-fitness region of the genome population shapes the convex region in the genotype space under sexual reproduction and selection (Fig. 4B).

Consequently, the fitness is expected to take on a single-humped structure around the center of the region and will decrease as the genotype moves toward the periphery of the high-fitness region (Fig. 4B). If the fit state is robust, the fitness is less sensitive to genetic change around the center than the periphery. This suggests that the fitness function is flat and becomes steeper when moving farther from the center (i.e. convex, single-humped functions). Thus, the fitness (phenotype) variance will be smaller than on the periphery around the center.

Now, consider the distribution of the homozygote group in a high-fitness region, whose genotype can be broadly located. This population contains individuals with low fitness and large phenotypic variance. Accordingly, a heterozygote group generated from a pair from the homozygote group is biased toward the center of the region. Therefore, the distribution of the heterozygote group is expected to have higher fitness and lower variance than that of the homozygote group, resulting in fitness- and variance-related heterosis. This picture is consistent with the observed correlation between heterosis and differential gene expression.

Thus, we expected the fitness landscape to be single-humped with a flatter top (i.e. convex) around the evolved genome population and examined the validity of this expectation in our model. First, we computed the distance between the two genome matrices $\sqrt{\sum _{i,j}(J^{\lt k\gt }_{ij}-J^{\lt l\gt }_{ij})^2}$ for the two genomes (*k, l*) in the population of the 999th generation. Among the combinations (*k, l*), we chose combinations of homozygotes (*K, L*) with the largest distance between genome matrices $\sqrt{\sum _{i,j}(J^{\lt K\gt }_{ij}-J^{\lt L\gt }_{ij})^2}$. Next, we produced 100 matrices corresponding to the internal division of $J^{\lt K\gt }_{ij}$ and $J^{\lt L\gt }_{ij}$ (i.e. $sJ^{\lt K\gt }_{ij}+(1-s)J^{\lt L\gt }_{ij}$) by changing *s* in a step-by-step manner in the range 0 ≤ *s* ≤ 1. We then computed the mean fitness for homozygotes $sJ^{\lt K\gt }_{ij}+(1-s)J^{\lt L\gt }_{ij},sJ^{\lt K\gt }_{ij}+(1-s)J^{\lt L\gt }_{ij}$ over 100 matrices of each division point given by *s*.

**Fig. 5.**
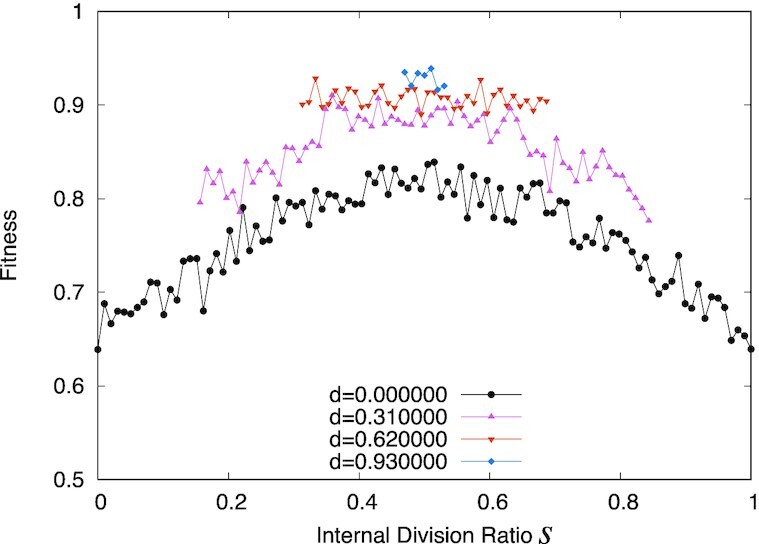
Fitness according to changes along a given genome pair, parametrized by *s*, where the internal division genome matrix is given by $sJ^{\lt K\gt }_{ij}+(1-s)J^{\lt L\gt }_{ij}$ between genome pairs (*K, L*). This was chosen so that the distance between genome matrices was the maximum value. The values at *x* = 0, 1 can be much lower because the homozygotes at the edge of the region accumulate too many mutations. The horizontal axis represents the internal division parameter *s*. The average fitness over 300 realizations is plotted for μ = 1.0 × 10^−4^ and σ = 1.0 × 10^−4^. Black curves are homozygotes. Here, we introduced the heterozygote $(s-\frac{d}{2})J^{\lt K\gt }_{ij}+(1-s+\frac{d}{2})J^{\lt L\gt }_{ij}$, $(s+\frac{d}{2})J^{\lt K\gt }_{ij}+(1-s-\frac{d}{2})J^{\lt L\gt }_{ij}$ with heterozygosity *d*. Pink, orange, and blue curves correspond to *d* = 0.31, *d* = 0.62, and *d* = 0.93 heterozygotes, respectively. The data for large *d* are plotted for a narrower range in *s*, because we used two genomes in the internal division from 0 to 1 for generating the heterozygotes.

As shown in Fig. 5, the fitness function of homozygotes exhibited a single-humped structure against the internal division parameter *s* and had a flatter landscape around the top, as predicted by our proposed explanation. As this type of fitness function is not observed in random matrices *J_ij_* (corresponding to the 0th generation), we suggest that it was acquired by evolution with sexual reproduction and selection. We did not assume this single-humped fitness function, but rather robustness in the fitness function under sexual reproduction might have caused the result.

The above estimate of the fitness landscape is based on the homozygote diploid ($sJ^{\lt K\gt }_{ij}+(1-s)J^{\lt L\gt }_{ij}$ and $sJ^{\lt K\gt }_{ij}+(1-s)J^{\lt L\gt }_{ij}$; i.e. haploid). To further analyze the fitness by heterozygotes, we introduced the heterozygote $(s-\frac{d}{2})J^{\lt K\gt }_{ij}+(1-s+\frac{d}{2})J^{\lt L\gt }_{ij}$, $(s+\frac{d}{2})J^{\lt K\gt }_{ij}+(1-s-\frac{d}{2})J^{\lt L\gt }_{ij}$ with heterozygosity *d* for each heterozygote with given *s*(Those heterozygotes are only placed between $\frac{d}{2}$ and $1-\frac{d}{2}$ because we used two genomes in the internal division from 0 to 1 for generating the heterozygotes). As shown in Fig. 5, the fitness with heterozygosity *d* > 0 was higher than that of the homozygotes(*d* = 0) with *s*; that is, the larger heterozygosity *d* implies a further increase of fitness. The fitness of such heterozygotes also exhibited a single-humped, convex function.

In Fig. 5, one can see two types of heterosis as in genome distribution and heterozygosity. First, even in the homozygote series(*d* = 0), the homozygote (haploid) at a middle internal division genome can achieve higher fitness than those at the edges. For example, the homozygote at the internal division point between *s* = 0.2(*w* ≈ 0.75) and *s* = 0.8(*w* ≈ 0.65) shows higher fitness (*s* = 0.5, *w* ≈ 0.85) than two. This implies that the event corresponding to heterosis can occur even in a complete homozygote or haploid population if the single-humped fitness function is achieved.

Second, interestingly, the heterozygosity further raises the fitness, as confirmed by including the above-mentioned heterozygosity parameter. For the heterozygosity *d* = 0.31, almost all heterozygotes showed higher fitness values than those of the homozygotes, whereas a single-humped fitness function was also obtained (see Fig. 5). The heterozygote advantage was further increased along with the increase in heterozygosity, as shown in the plots in Fig. 5 for *d* = 0.62, 0.93. These results imply the existence of a heterozygote advantage beyond the effect of the removal of deleterious mutations. At the top of the single-humped fitness function of homozygotes, the offspring of the highest homozygotes (i.e. the ”best-parents”) can achieve higher fitness than the original homozygotes, as is also discussed in the overdominance model.

Here, generally, even if the population’s average fitness stays constant, there are still variations in genomes, and some heterozygotes with lower fitness may exist, which are placed in the periphery of the convex region. If we make them homozygotes, the fitness will be lower than that of the heterozygote. Here, we chose a pair from the opposite side of the periphery to demonstrate the relevance of the convexity to robustness. Thus, the fitness of the heterozygote from the opposite side of the periphery of fitness is higher than that of the homozygote, which provides one cause for heterosis.

To confirm this scenario, we took the individuals with the worst 5% fitness within the evolved population and generated their offspring either by sexual reproduction without mutation or by mutation only (without sexual reproduction). On the average, the sexual reproduction only showed the average fitness at 0.66, much higher than that by the mutation only, which was 0.35, and the average fitness of the original worst 5% population, which was 0.26. The distributions of fitness of those three cases are shown in Figure S5 (Supplementary Material). When we make the offspring through sexual reproduction from all individuals in the steady-state population, whose average fitness was 0.844, whereas the average fitness of sexually reproduced individuals, 0.843, was slightly lower than that but distinctively higher than that of asexually reproduced ones, 0.836. These results are consistent with the picture in Fig. 4.

### Comparison with the case without sexual reproduction (selfing)

Comparison with selfing is also relevant to conclude that the observed heterosis results from sexual reproduction. Here, we examined the case with a single parent, without sexual reproduction, which can be regarded as corresponding to selfing. A total of two genetic modes, with and without recombination, were numerically investigated. (See the Material and Methods for the definition of selfing genetic modes.)

**Fig. 6.**
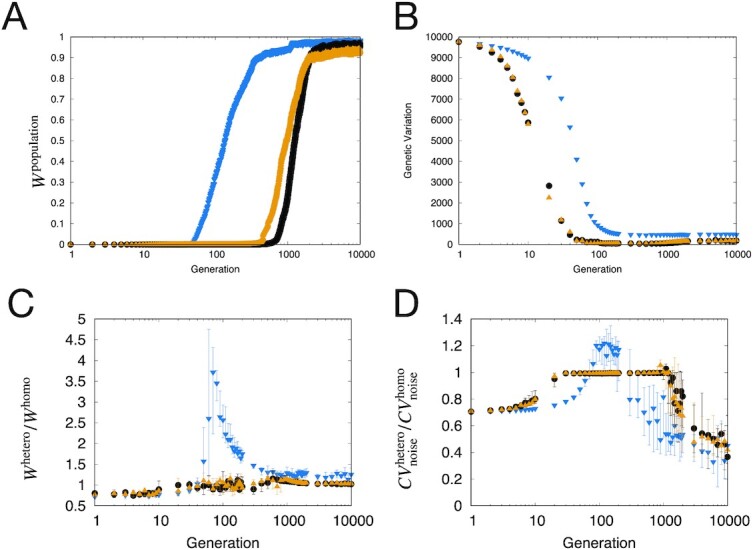
Evolution of heterosis, fitness, and genetic variation. In all graphs, blue points result from evolution with meiosis in the genetic algorithm, black points are selfing without recombination, and orange points indicate selfing with recombination. The mutation rate per edge was μ = 1.0 × 10^−4^ and the noise strength was σ = 1.0 × 10^−4^. The points represent the average of 30 realizations, and error bars range from the first to the third quantiles. All horizontal axes are shown on the logarithmic scale. (A) Fitness increases throughout evolution. *W*^population^ is the average fitness of the evolving population. The meiosis population evolved faster than the other two selfing populations, but the fitness of all three populations reached values larger than 0.9. (B) Change in the genetic variation in the population throughout evolution. Genetic variation is defined as the sum of the variance in specific *J_ij_* elements in the population. The meiosis population showed higher genetic variation than the other two selfing populations. (C) Change in the degree of fitness-related heterosis *W*^hetero^/*W*^homo^ throughout evolution. All three genetic modes showed *W*^hetero^/*W*^homo^ > 1, but that of meiosis was larger than that with selfing. (D) Change in the degree of fitness-related heterosis $CV^\mathrm{hetero}_\mathrm{noise}/CV^\mathrm{homo}_\mathrm{noise}$ throughout evolution. All three genetic modes showed $CV^\mathrm{hetero}_\mathrm{noise}/CV^\mathrm{homo}_\mathrm{noise}\lt 1$ values after the increase of fitness, but those with meiosis were smaller than those with selfing.

The evolvability by both selfing populations was slower than that of the case with sexual reproduction involving meiosis (Fig.6A). This is consistent with earlier studies (36, 58). Genetic variation decreased throughout evolution in all three genetic modes, as shown in Fig. 6(B); however, the result with meiosis showed higher genetic variation than that of the other two cases. For the selfing case without sexual reproduction, *W*^hetero^/*W*^homo^ was lower and $CV^\mathrm{hetero}_\mathrm{noise}/CV^\mathrm{homo}_\mathrm{noise}$ was higher than those of the other cases, as shown in Figs 6(C) and (D). Hence, sexual reproduction with meiosis increases the degree of heterosis compared with selfing, stronger heterosis, or inbreeding depression.

For reference, we also conducted a heterosis test in the neutral evolution scenario to investigate the selection effect on our heterosis result, which had the fitness function of *w* = 1 for any genotypes and phenotypes. The result of the neutral simulation in Figure S6 (Supplementary Material) showed that the variance-related heterosis was still achieved, whereas the ratio $CV^\mathrm{hetero}_\mathrm{noise}/CV^\mathrm{homo}_\mathrm{noise}$ was larger than that obtained in the case under selection. Comparing Fig. 1(D) with Figure S6 (Supplementary Material), the first increase of $CV^\mathrm{hetero}_\mathrm{noise}/CV^\mathrm{homo}_\mathrm{noise}$ would be a result of genetic drift, while the subsequent decrease would be due to selection.

### Case of population separation

The numerical procedure we adopted to compare the homozygote and heterozygote groups was based on hybridization among inbreeding individuals. Such a procedure can be achieved in some laboratory experiments by using yeast (59–61). In contrast, most experimental studies on heterosis or inbreeding depression adopt population separation and inbreeding among divided populations. Hence, we also conducted the simulation using such population separation in order to confirm the validity of the results obtained so far. To be specific, we conducted the following simulation to evolve the population. First, we divided the population of 100 individuals into two populations of 50 each. Second, we conducted the same evolutionary simulation with the same fitness conditions over 100 generations, under the constraint that the 50 individuals in each divided population reproduce, thereby keeping the same number of individuals in the divided populations. Third, hybrids between the two separated populations were generated to compare with each inbreeding population. The fitness and noise-induced CV were then compared between the inbred and hybrid populations (*W*^inbred^, $W^\mathrm{hybrid},CV^\mathrm{inbred}_\mathrm{noise}$, $CV^\mathrm{hybrid}_\mathrm{noise}$).

**Fig. 7.**
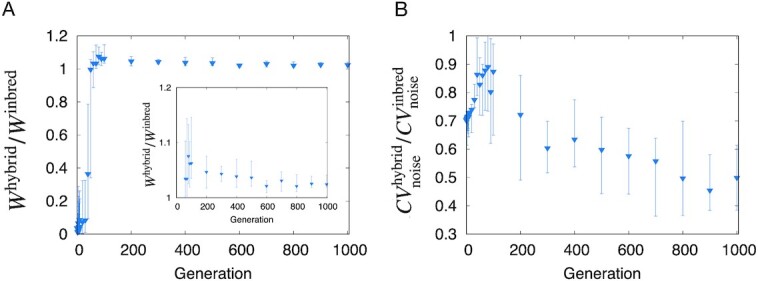
Evolution of heterosis in the population separation simulation. (Parameters and conditions were that same as those in Fig.1(A) and (D).) (A) Change in the degree of fitness-related heterosis *W*^hybrid^/*W*^inbred^ throughout evolution. *W*^hybrid^/*W*^inbred^ increased initially and then decreased thereafter. Relaxed *W*^hybrid^/*W*^inbred^ value was near one but *W*^hybrid^/*W*^inbred^ > 1 was maintained. (B) Evolution of the median value of the degree of variance-related heterosis $CV^\mathrm{hybrid}_\mathrm{noise}/CV^\mathrm{inbred}_\mathrm{noise}$. The $CV^\mathrm{hybrid}_\mathrm{noise}/CV^\mathrm{inbred}_\mathrm{noise}$ increased initially and then decreased through evolution, reaching a median value of approximately 0.5.

Figure7  shows the evolution of heterosis in the population separation simulation. The degree of fitness-related heterosis *W*^hybrid^/*W*^inbred^ after evolution satisfied *W*^hybrid^/*W*^inbred^ > 1, corresponding to fitness-related heterosis. One might still argue that *W*^hybrid^/*W*^inbred^ was lower than shown in Fig. 7(A). This would be due to the long-term selection after population separation, which increased the fitness of the inbred population. In fact, *W*^hybrid^/*W*^inbred^ was much larger (∼ 1.1) before it reached the stationary value. Moreover, the variance-related heterosis after evolution was also satisfied, as $CV^\mathrm{hybrid}_\mathrm{noise}/CV^\mathrm{inbred}_\mathrm{noise} \approx 0.5\lt 1$, even though the value was slightly larger than that obtained by the previous procedure, ≈0.4. To sum, the heterosis was also confirmed in the population separation procedure.

## Discussion

In the present study, we employed numerical evolution of the diploid-GRN to demonstrate heterosis related to fitness and phenotypic CV and its correlation with differential gene expression, and further compared the simulation results with previously reported measurement data. First, heterozygote groups exhibited higher fitness than homozygote groups, indicating heterosis (or inbreeding depression). Phenotypic CV due to noise was also lower in the heterozygote group than in the homozygote group, indicating variance-related heterosis. The numerically obtained distribution of the ratio of the CV of the homozygote group to that of the heterozygote group showed similarities with that of the previous measurement data (41). Heterosis exhibited a negative correlation with expression pattern dominance and a positive correlation with differential gene expression. We then proposed a possible explanation for these results according to the fitness landscape, which exhibits a convex shape if high fitness is maintained under sexual reproduction and selection. We also conducted selfing and population separation simulations to confirm the validity of our fitness and variance-related heterosis results.

Here, we remark on variance-related heterosis. The traditional quantitative genetics theory explained heterosis with respect to the total phenotypic variance based on the correlation variance; however, there was a discrepancy between the theory and the observed distribution in (41). Furthermore, we compared noise-induced phenotypic variance *V*_noise_, which corresponds to the inverse of noise robustness. The heterosis concerning noise-induced variance has scarcely been studied, even though such noise robustness has recently attracted substantial attention in the context of research on homeostasis and cell state selection (26,43–45, 62); thus, heterosis should also be reexamined in light of noise robustness. Recall that even a random network or selfing can produce considerable variance-related heterosis to some degree; however, sexual reproduction shows quantitative deviation from these scenarios.

The ratio of *CV*_fitness_, which contains the genetic variation, showed similarity to the experimental data by Phelan and Austed. Here, however, *CV*_noise_ occupied the major part of the total phenotypic CV. These results imply that noise robustness and genetic variation would contribute to the variance-related heterosis. Nevertheless, further experiments for given species under fixed environmental conditions will be needed to make a further quantitative comparison, together with ploidy manipulation and direct measurement of the noise. For example, such experiments have been performed in yeast (59–61), in which the GRN has already been analyzed (63–65) in the heterosis context.

We also found a positive correlation between differential gene expression and heterosis. This correlation has also been discussed previously (19–22), whereas the molecular mechanism contributing to heterosis remains unclear (66). According to the present study, such correlation arises from phenotypic robustness due to interactions among genes through the GRN.

We proposed the convex fitness landscape and single-humped fitness function shaped by evolution to explain our results on heterosis. Under evolution with sexual reproduction, it is necessary to create a population of genomes in which the fitness of the offspring is not reduced from those of the parents. A population with a convex fitness landscape and single-humped fitness function will satisfy such a postulate. This viewpoint was also discussed in the context of basic quantitative genetics, in which the fitness landscape or function for a given genome distribution is provided as such. In contrast, we did not explicitly define such a fitness landscape. The fitness landscape does not exist in the first generation but is rather shaped by evolution under sexual reproduction. Furthermore, for the case of selfing, the observed heterosis was much weaker.

The single-humped fitness function leads to an increase in robustness against noise. This explanation based on the fitness landscape and adaptive walk may be similar to the argument proposed on the heterozygote advantages by Fisher’s geometric model (67). However, in the present study, the evolution of robustness to noise in gene expression dynamics shaped such a convex fitness landscape.

The convex fitness landscape and single-humped fitness function may suggest a possible relationship between the dominance and overdominance models. At the periphery of the single-humped fitness function, the dominance model to eliminate the deleterious mutation effect would be valid for explaining heterosis. Around the center of the function, the overdominance model would account for the heterosis result. In the present study, however, further heterozygote superiority arose from the interaction between different GRNs.

We confirmed our result by simulating the divided populations, as adopted in standard heterosis measurements. In the future, we need to examine the dependence on the division ratio or the length of inbreeding to compare with the analysis based on a population genetics approach. In the discussion of heterosis based on gene regulation, the importance of large-scale rewiring in GRNs has been highlighted (17). In fact, in our simulation, heterosis was strengthened under a high mutation rate during evolution. Heterosis, at least to some degree, can be understood by phenotypic robustness under the presence of large-scale rewiring in the GRN by sexual recombination.

To conclude, we elucidated the evolution of heterosis in a system of interacting genes by noting the relevance of robustness to noise as well as sexual recombination (68) with meiosis of genomes (69). This evolution of heterosis also implies the advantage of sexual reproduction, which goes beyond the standard concept of Muller’s ratchet (70) due to the removal of deleterious mutants. Alternatively, the explanation by differential gene expression presented in this study suggests that heterozygosity in diploid organisms goes beyond the range of homozygote phenotypes due to the interactions between the two genomes, which creates a convex fitness landscape through evolution. As the present evolution model does not need to assume inbreeding as adopted in standard heterosis studies, this study will shed novel light on the evolution of heterosis that is applicable to field and laboratory experiments.

## Materials and Methods

### Derivation of diploid-GRN model

Let us define $y^m_i(t)$ as the concentration of mRNA *i* from each chromosome (*m* = 1, 2) and *x_i_*(*t*) as the concentration of the corresponding protein in a cell. Note that the proteins are synthesized from both chromosomes, where mutations or recombination are mainly introduced in the promoter or enhancer region; thus, the superscript *m* in *x_i_*(*t*) is not required. By replicating the regulation in a diploid cell by $J^{(m)}_{ ij}$ (*m* = 1, 2) for each gene, we obtain

$$\begin{eqnarray*}
y^{m}_i(t+1) = \frac{1}{2}f[ \sum _{j=1}^N J^{(m)}_{ij}(x_j(t)-\theta )]\ (m=1,2),
\end{eqnarray*}
$$

where θ( = 0.5) is the threshold of expression, which makes the states *x* = 0 and *x* = 1 symmetric for simplicity. As protein *i* is synthesized from the corresponding mRNA, the total expression level is

$$\begin{eqnarray*}
x_i(t+1)=\sum _{m=1}^{2} y^m_i(t+1).
\end{eqnarray*}
$$

Therefore,

$$\begin{eqnarray*}
x_i(t+1)=\frac{1}{2}\sum _{m=1}^{2} f[ \sum _{j=1}^N J^{(m)}_{ij}(x_j(t)-\theta )].
\end{eqnarray*}
$$

### Details of the evolutionary simulation

Fitness is computed as follows. After a sufficient time, *T*, the expression level *x_i_*(*t*) reaches a stationary state. Here, we define the phenotype as the time average of *x_i_*(*t*) for the last 10 steps, $\bar{x_ i}=\frac{1}{10}\sum _{t=T-9}^{T}x_i(t)$. The fitness is defined by this average $\bar{x_ i}$ for the target genes *i* = 1,..., *M*( < *N*)(Positive interactions between target genes can achieve this target pattern. However, there are nontarget genes in the network, which may affect the expression of target genes (the path ratio of the GRN was set to 0.5)). By setting the maximum fitness for $\bar{x_i}-1=0$ (*i* = 1,..., *M*), the absolute fitness is defined as $\exp [S(-\sum ^{M}_{i=1} |\bar{x_i}-1|)]=\exp [S\sum ^{M}_{i=1}\bar{x_i}]\exp [-SM]$. Then, the relative fitness *w* is given by $w=\exp [S\sum ^{M}_{i=1}\bar{x_i}]$ and the probability that an individual having $\bar{x_i}$ is selected is proportional to *w*. In some cases, the expression pattern *x_i_*(*t*) oscillates over time and cannot reach a stable state; however, this is rare. Moreover, after evolution, such a case is not observed because a stable expression pattern $\bar{x_i}=1$ (*i* = 1,..., *M*) can be more advantageous than an oscillatory state under this fitness definition.

Note that the target pattern for fitness is assigned only for some genes(*i* = 1,..., *M*), so that there is no selection pressure for the expression level of most genes (*i* = *M* + 1,..., *N*). In this simulation, *N* = 100 and *M* = 10. Furthermore, the threshold *θ* = 0.5 so that the states *x_i_*(*T*) = 0 and *x_i_*(*T*) = 1 are symmetric, which are neutral in the nontarget genes (*i* = *M* + 1,..., *N*)(The nontarget genes are neutral as is the case for fitness. However, for each network, the nontarget genes can affect the expression levels of target genes through regulatory interactions, which are also changed by mutation or recombination.).

We used the following meiosis-like genetic algorithm for the evolutionary simulation. Two distinct individuals *k*_1_ and *k*_2_ are selected as parents with probabilities of $w_{k_1}/W$ and $w_{k_2}/W$, from which the next generation is produced according to the above fitness definition (*k*_1_ ≠ *k*_2_), where $W=\sum _{k=1}^K w_k$ is the sum of all fitness values in the population. To introduce meiosis, we adopted the following procedure. A single gamete is generated by mixing $[J^{(1)}_{ij}]_{k_1}$ and $[J^{(2)}_{ij}]_{k_1}$ ($[J^{(1)}_{ij}]_{k_2}$ and $[J^{(2)}_{ij}]_{k_2}$) via row vectors with equal probabilities to produce a new genome *n*, where $[J^{(1)}_{ij}]_{n}$ ($[J^{(2)}_{ij}]_{n}$).

We also conducted two other procedures corresponding to selfing genetic modes for reference. In these genetic modes, there is one parent that is chosen by the probability *w_k_*/*W*. In the first genetic mode, selfing without recombination, $[J^{(1)}_{ij}]_{k}$ or $[J^{(2)}_{ij}]_{k}$ are copied as the one of the new diploid genome *n*. A total of two new genomes can be the copies of the same parental genome. In the second genetic mode, selfing with recombination, $[J^{(1)}_{ij}]_{k}$ and $[J^{(2)}_{ij}]_{k}$ are mixed via row vectors with equal probabilities to produce a new genome *n*. We have adopted this setting of recombination between the two genomes in one parent to clarify the role of genome mixing between two parents. This setting corresponds to selfing.

The simulation was then performed by changing the mutation rate (μ) in the range [10^−5^, 10^−2^] and the standard deviation of the noise (σ) in the range [5 × 10^−4^, 10^−1^]. The number of genes (*N*) was set to 100. The ratio of nonzero elements in $J^{(m)}_{ij}$ was set to 0.5 so that some $J^{(m)}_{ij}$ elements changed to zero after mutation or recombination to maintain this ratio. The selection pressure, *S*, was set to 2 and the number of target genes (*M*) was 10. The relaxation time (*T*) to examine fitness was 30. The total number of individuals (*K*) included in the study was 100. Out of the total number of genes, 90 are not initially expressed.

### Definition of *CV*_noise_

*CV*_noise_ is the phenotypic CV generated by noise over isogenic individuals with a particular genotype. It is computed by running simulations of gene expression dynamics with stochastic noise multiple times for the individual under the same genotype. For a given diploid individual *k* with $J^{(1)}_{ij}$ and $J^{(2)}_{ij}$, $\bar{x_i}$ after relaxation is computed under a given noise magnitude σ( ≠ 0). After repeating this process *L* times and obtaining $\bar{x_i}$ for each run, we measured the variance of the expression pattern $\bar{x_i}$. The CV $cv^k_\mathrm{noise}$ for an individual *k* was computed using the average of the CV over all genes(*N* = 100) and realizations (*L* = 100). *CV*_noise_ was computed by the average of $cv^k_\mathrm{noise}$ over the individuals for a given genome group.

### Pattern dominance: definition and calculation

First, we extracted 2*K* genomes $J^{\lt k\gt }_{ij}(k=1....2K)$ from *K* diploid individuals in the evolutionary simulation. We then made a complete copy for each 2*K* genome, creating a population of 2*K* complete homozygotes (e.g. $J^{\lt k\gt }_{ij}J^{\lt k\gt }_{ij}$). Then, we computed the expression dynamics in each of the 2*K* complete homozygotes to obtain the expression pattern $x^{AA}_i$. Next, from the 2*K* genomes, two $J^{\lt A\gt }_{ij}$ and $J^{\lt B\gt }_{ij}$ are randomly selected to create a diploid cell consisting of two heterozygous genomes. From the expression dynamics of the heterozygous genomes, the expression pattern of heterozygous $x^{AB}_i$ is obtained.

We could then compare the expression of genes that are differentially expressed between two such homozygotes: those with $x^{AA}_i\ne x^{BB}_i$. By taking into account that *x_i_* is real, the (in)equality *x* ∼ *y* (*x* ≠ *y*) is defined if |*x* − *y*| < 0.1 or |*x* − *y*| > 0.1, as a numerical procedure. For most cases, *x_i_* takes the value of ∼1 or ∼0; however, the definition is basically the same whether the genes are expressed(∼1) or not(∼0). The number of *i* genes with $x^{AA}_i\ne x^{BB}_i$ is defined as $N^{AA,BB}_\mathrm{dif}$. Next, the number of $x^{AB}_i \simeq x^{AA}_i$ ($x^{AB}_i \simeq x^{BB}_i$) is defined as $N^{AB}_\mathrm{A}$ ($N^{AB}_\mathrm{B}$). Then, the degree of dominance is given by $\max (N^{AB }_\mathrm{A}/N^{AA,BB}_\mathrm{dif}, N^{AB }_\mathrm{B}/N^{AA,BB}_\mathrm{dif})$.

In the current model, the expression level of each gene *x_i_* is almost always very close to zero or one. Therefore, if only one gene is differentially expressed between the two homozygotes, the calculated dominance will always be one. To remove this bias, we calculated the pattern dominance only for homozygote $x^{AA}_i$ and $x^{BB}_i$ pairs in which more than 10 genes are differentially expressed. For reference, we also show the gene expression pattern of the homozygote and heterozygote groups in the Supplementary Material.

### Differential gene expression: definition and calculation

**Fig. 8.**
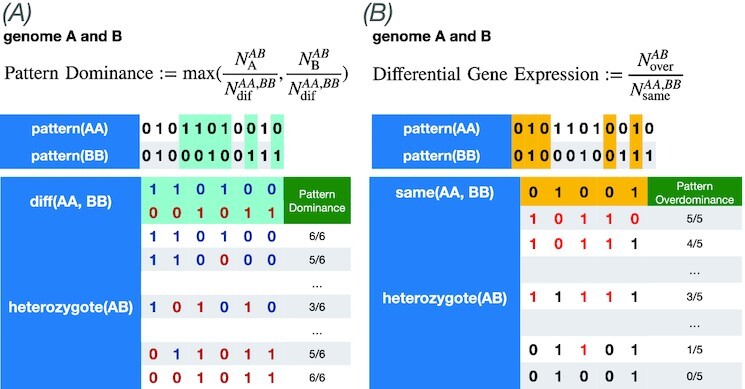
Definition and calculation of pattern dominance and differential gene expression. (A) Pattern dominance: first, we compute the expression patterns of homozygotes *AA* and *BB*. Next, the expression pattern is computed by making the heterozygote *AB*. We then calculate the percentage of the expression pattern obtained from the heterozygote that matches only one of *AA* or *BB* for the differentially expressed genes among the two homozygotes, focusing on the differentially expressed genes in the two homozygotes (blue). The pattern dominance is then computed as a larger percentage of matches between $x^{AB}_i$ and $x^{AA}_i$ or between $x^{AB}_i$ and $x^{BB}_i$. (B) Differential gene expression: for a gene showing the same expression in the two homozygotes (blue), we calculate the percentage of the expression pattern obtained from the heterozygote that is different from that of *AA* and *BB*. Then, the differential gene expression is computed as the percentage of $x^{AB}_i \ne x^{AA}_i(= x^{BB}_i)$.

In contrast to pattern dominance that focuses on the expression patterns of genes that differ between homozygotes of the two genomes, differential gene expression focuses on genes expressed in the same manner between the two genomes. Let $N^{AA,BB}_\mathrm{same}$ be the number of genes with the same expression ($|x^{AA}_i-x^{BB}_i|\lt 0.1$) as the two homozygotes, and $N^{AB}_\mathrm{over}$ be the number of genes in the heterozygote *AB* that are expressed differently($|x^{AA}_i-x^{AB}_i|\gt 0.1$ or $|x^{BB}_i-x^{AB}_i|\gt 0.1$) from those in the homozygotes *AA* and *BB*. $N^{AB}_\mathrm{over}/N^{AA,BB}_\mathrm{same}$ is then defined as the degree of differential gene expression (Fig.8).

## Supplementary Material

pgac097_Supplemental_File
