# Supplementary material for: Heterosis of fitness and phenotypic variance in the evolution of a diploid gene regulatory network
Source: PNAS Nexus. 2022 Jun 29;1(3):pgac097. doi: 10.1093/pnasnexus/pgac097 (PMC9896930; doi:10.1093/pnasnexus/pgac097)
Supplement: pgac097_Supplemental_File [file pgac097_supplemental_file.pdf]

Supplementary Information for

Heterosis of Fitness and Phenotypic Variance in the Evolution of a Diploid Gene Regulatory Network

Kenji Okubo, Kunihiko Kaneko

Kunihiko Kaneko.
E-mail: kaneko@complex.c.u-tokyo.ac.jp

This PDF file includes:

Supplementary text
Figs. S1 to S6 (not allowed for Brief Reports)

Supporting Information Text

Estimation of $V_{\text{noise}}^{\text{homo}} \approx 0.250$ and $V_{\text{noise}}^{\text{hetero}} \approx 0.125$ in random networks

Here, we explain $V_{\text{noise}}^{\text{homo}} \approx 0.250$ and $V_{\text{noise}}^{\text{hetero}} \approx 0.125$ in random networks, which correspond to the population of the 0th generation in our simulation.

First, recall that the dynamics of $x_i(t)$ are given by

$$x_i(t+1) = f[\sum_{j=1}^{N} J_{ij}^{(1)} x_j(t)] + f[\sum_{j=1}^{N} J_{ij}^{(2)} x_j(t)] + \sqrt{x}\eta(0,\sigma). \quad [1]$$

We assume that, in a random network, the final state (fixed point) is randomly determined. In this case, because homozygotes have the same J_{ij}, they can only take on two states: $(f[\sum_{j=1}^{N} J_{ij}^{(1)} x_j(t)], f[\sum_{j=1}^{N} J_{ij}^{(2)} x_j(t)]) = (0,0),(1,1)$. Therefore, the normalized expression level is randomly zero or one. The frequencies of the expression levels 0, 0.5, and 1 are 0.5, 0, and 0.5, respectively. Conversely, in heterozygotes, the expression does not have to be identical in the two genomes; thus, there are four possible states: $(f[\sum_{j=1}^{N} J_{ij}^{(1)} x_j(t)], f[\sum_{j=1}^{N} J_{ij}^{(2)} x_j(t)]) = (0,0),(0,1),(1,0),(1,1)$. The frequencies of the expression levels 0, 0.5, and 1 are 0.25, 0.5, and 0.25, respectively. Therefore, we obtain $V_{\text{noise}}^{\text{homo}} = 0.250$ and $V_{\text{noise}}^{\text{hetero}} = 0.125$.

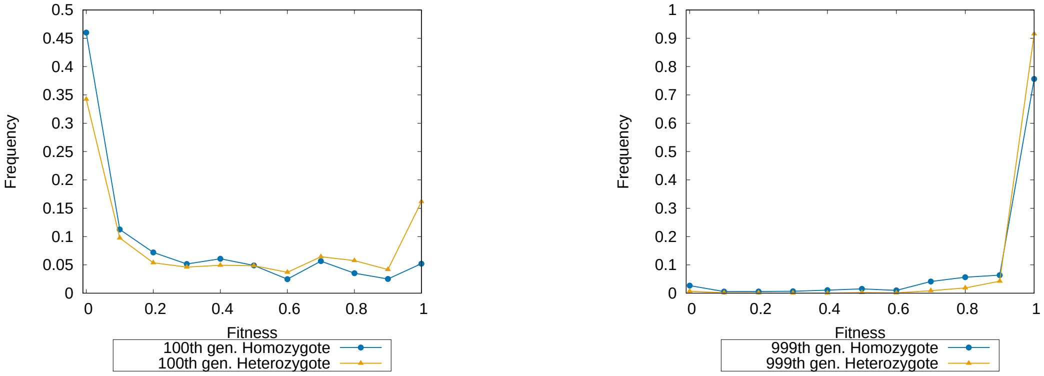

Fig. S1. Distributions of W^{hetero} and W^{homo} at evolutionary generation 100 and 999. The mutation rate per edge is $\mu = 3 \times 10^{-5}$ and the noise strength is $\sigma = 5 \times 10^{-4}$. The bin is 0.1. The distribution is computed for 50 realizations. At the 0th generation, a random network cannot achieve an expression level of one in the target gene; both W^{homo} and W^{hetero} are concentrated at zero. (Therefore, these data are not shown.) At the 100th generation, some W^{hetero} exhibit larger values than W^{hetero}. At the 999th generation (after evolution), W^{hetero} reaches the maximum fitness of close to one more frequently than W^{homo}. Here, $< W^{\text{homo}} >< < W^{\text{hetero}} >$.

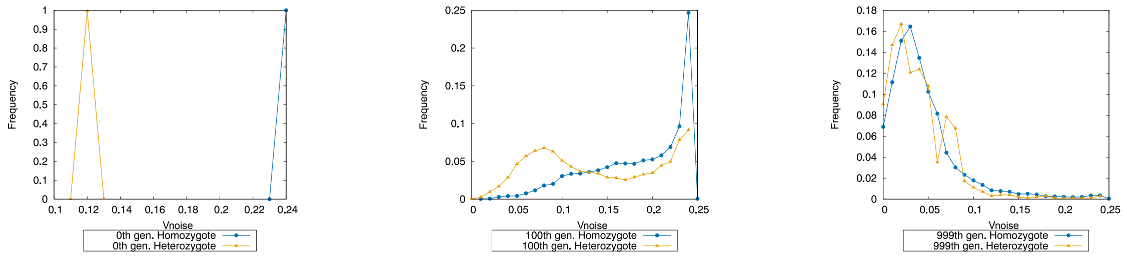

Fig. S2. Distributions of $V_{\mathrm{noise}}^{\mathrm{homo}}$ and $V_{\mathrm{noise}}^{\mathrm{hetero}}$. The mutation rate per edge is $\mu = 3 \times 10^{-5}$ and the noise strength is $\sigma = 5 \times 10^{-4}$. The bin is 0.01. The distribution is computed for 50 realizations. At the 0th generation, the distribution is concentrated at $V_{\mathrm{noise}}^{\mathrm{homo}} \approx 0.250$ and $V_{\mathrm{noise}}^{\mathrm{hetero}} \approx 0.125$, as explained by the random networks. At the 100th generation, the distribution of $V_{\mathrm{noise}}^{\mathrm{hetero}}$ has a much smaller peak value than $V_{\mathrm{noise}}^{\mathrm{homo}}$. Throughout evolution, both $V_{\mathrm{noise}}^{\mathrm{homo}}$ and $V_{\mathrm{noise}}^{\mathrm{hetero}}$ decrease and the peaks of the distributions are shifted to smaller values. The relationship $V_{\mathrm{noise}}^{\mathrm{hetero}} < V_{\mathrm{noise}}^{\mathrm{homo}}$ is maintained. This implies that the increase in robustness is more prominent for heterozygotes, leading to heterosis related to phenotypic variance.

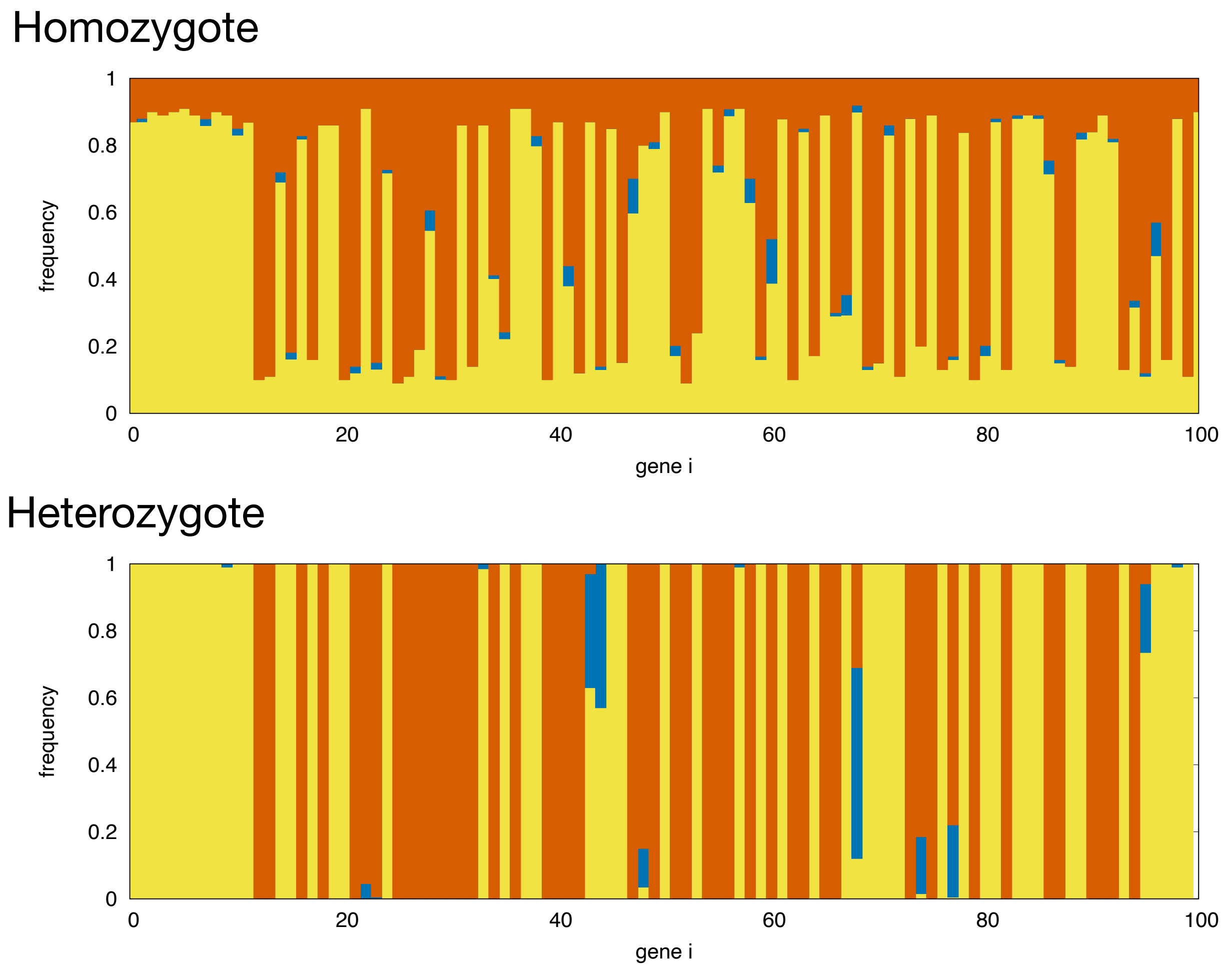

Fig. S3. Frequency of the expression patterns in the homozygote group and heterozygote group. Yellow, blue, and orange show the ratio of expression $x_i < 0.1$, $0.1 \le x_i \le 0.9$, and $x_i > 0.9$, respectively. The ratio is computed for the expression of each gene (not for all genes).

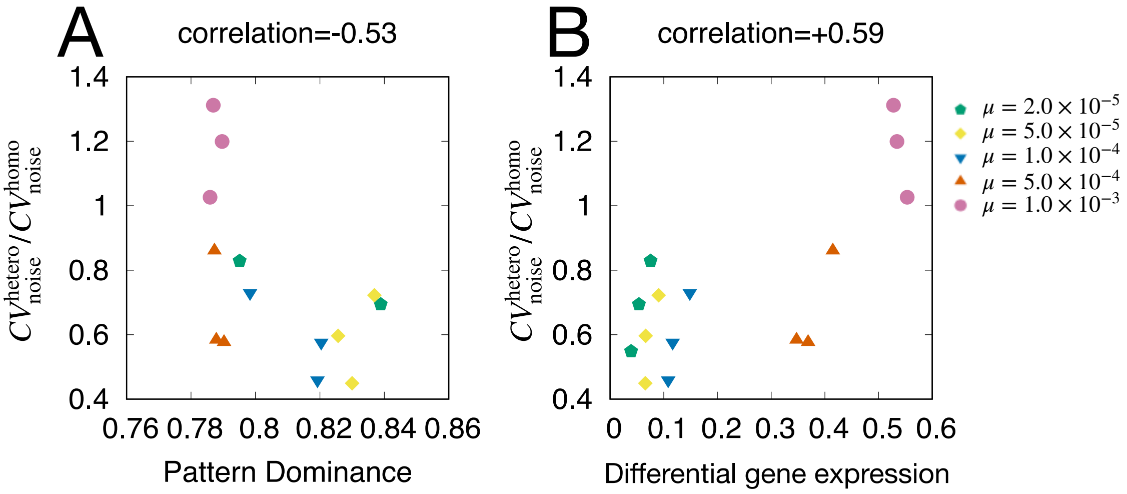

Fig. S4. Correlation between heterosis related to phenotypic variance and dominance (differential gene expression). (*A*) Correlation between pattern dominance and $CV_{\rm noise}^{\rm hetero}/CV_{\rm noise}^{\rm homo}$. (*B*) Correlation between differential gene expression and $CV_{\rm noise}^{\rm hetero}/CV_{\rm noise}^{\rm homo}$. Separate points in each mutation rate series represent different points of noise magnitude σ of $[1 \times 10^{-4}, 1 \times 10^{-2}]$. Points represent the average over 30 realizations, computed at the 10000th generation. Thus, heterosis related to phenotypic variance is achieved as the pattern dominance is acquired; however, the absolute value of the correlation coefficient is smaller than that obtained for heterosis related to fitness.

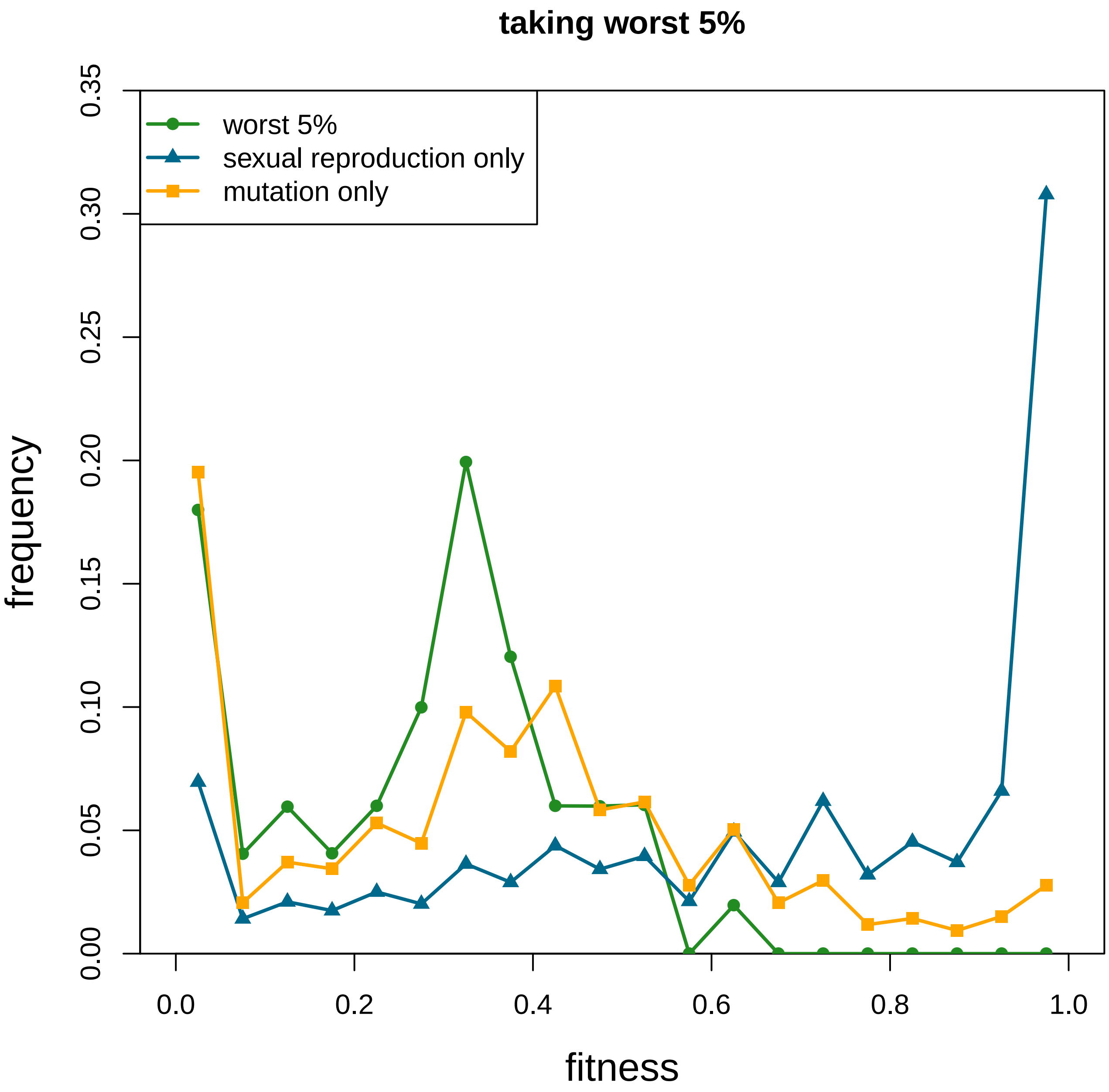

Fig. S5. The fitness distributions were generated from lower fitness parents. First, we took five individuals with the worst 5% fitness out of 100 individuals in the evolved population in the 2000th generation. Second, we generated offspring from them with sexual reproduction without mutation. Third, we generated the worst 5% individuals' offspring with asexual reproduction with the mutation only. The total number of individuals is 200 parents for 100 times in 10 realizations in the worst5% (200000 at total), 100 offspring for 100 times in 10 realizations in the sexual reproduction only (100000 at total), and the same number of the offspring with the mutation only. The mutation rate per edge is $\mu = 2.0 \times 10^{-3}$ and the noise strength is $\sigma = 0$. This histogram shows the frequency of fitness from the above three cases. The original (worst 5%) distribution is shown by green dots and lines, that by sexual reproduction only ones by blue triangles, and that by mutation only ones by orange squares. Compared to the worst 5% individuals, offspring by only sexual reproduction raised their fitness (this is a similar distribution to the original evolved population), and offspring by only mutation had much lower fitness (only slightly raised their fitness).

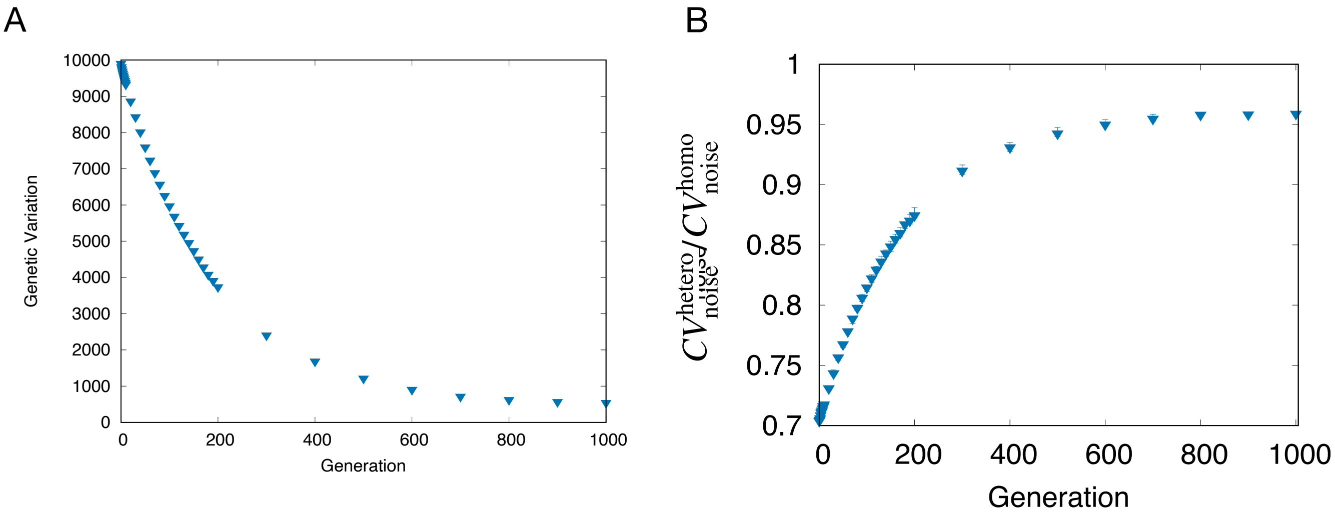

Fig. S6. The evolution of genetic variation (*A*) and $CV_{\text{noise}}^{\text{hetero}}/CV_{\text{noise}}^{\text{homo}}$ (*B*) in a neutral fitness context. The fitness function is set to $w = 1$ for any genotype and phenotype. At the first generation, the genetic variation was around 10,000 because we started the simulation from a random network. Along with a decrease in genetic variation by genetic drift, $CV_{\text{noise}}^{\text{hetero}}/CV_{\text{noise}}^{\text{homo}}$ increases and reaches a value of approximately 0.92. $CV_{\text{noise}}^{\text{hetero}}/CV_{\text{noise}}^{\text{homo}} < 1$ was maintained, but was larger than that under selection.
